# Influence of Potassium-Based Alkaline Electrolyzed Water on Hydration Process and the Properties of Cement-Based Materials with Fly Ash

**DOI:** 10.3390/ma14226956

**Published:** 2021-11-17

**Authors:** Zexin Yu, Zixi Xie, Tianyu Zhang, Gongbing Yue, Haibao Liu, Qiuyi Li, Liang Wang

**Affiliations:** 1 School of Architectural Engineering, Qingdao Agricultural University, Qingdao 266109, China; zexinyu666@163.com (Z.Y.); xzx1143017150@163.com (Z.X.); yuegongbing@163.com (G.Y.); 2 School of Civil Engineering, Qingdao University of Technology, Qingdao 266033, China; z17860423856@163.com (T.Z.); lhbhxw@163.com (H.L.)

**Keywords:** alkaline electrolyzed water, fly ash, microstructural methods, cement hydration, workability, mechanical properties, durability

## Abstract

Alkaline electrolyzed water, a kind of clean green water with excellent characteristics such as high activity, strong alkalinity, high ion penetrating ability, electrical charge, and good molecule adsorption, was significant to the resource utilization of industrial fly ash waste. This paper studies highly active potassium-based alkaline electrolyzed water’s impact, compared with ordinary water, on the cement hydration process using microstructural methods such as a hydration heat test, differential thermal analysis, X-ray diffraction (XRD) pattern, and Scanning electron microscope (SEM) image analysis. Fly ash cement-based materials were first prepared with alkaline electrolyzed water as the mixing water. The alkaline electrolyzed water’s influence on fly ash paste workability and the mechanical properties of fly ash mortar for varying fly ash proportions were ratified. Then alkaline electrolyzed water with the best pH value was selected to prepare fly ash concrete, and its durability was studied. The test results showed that it is feasible to increase the utilization rate of fly ash by using alkaline electrolyzed water. Furthermore, it promoted the process of cement hydration, increased the rate of the hydration reaction, and the promotion effect increased with the increase in pH value of the alkaline electrolyzed water, and also promoted the effective decomposition of the vitreous shell of fly ash to stimulate its early activity. Concurrent tests with ordinary water paste showed that the water requirement for normal consistency and setting time with alkaline electrolyzed water paste were significantly less. Alkaline electrolyzed water also solved the problem related to the low early strength of fly ash mortar. Furthermore, using alkaline electrolyzed water with an optimum pH value of 11.5 to prepare fly ash concrete effectively reduced concrete’s carbonation depth and carbonation rate and lessened the chloride ion migration coefficient.

## 1. Introduction

With the rapid development of the energy and electric power industries, the total fly ash (FA) emitted by coal-fired thermal power plants has increased yearly. At present, most of the FA gets buried in landfills and via other traditional methods. However, much delayed FA has caused a colossal waste of resources and brought severe environmental pollution [1,2,3]. Therefore, as FA is the leading industrial solid waste, its efficient resource reuse is in line with the technical and economic policy of turning waste into treasure and meeting global sustainable development requirements [4,5,6,7,8].

Nowadays, the preparation of high-performance concrete with FA as an active mineral admixture is the leading research direction for the high-efficiency worldwide utilization of FA. Its pozzolanic effect and micro-filling effect effectively has improved concrete and cementitious materials [9,10,11,12,13]. However, the metastable vitreous structure in FA has high chemical stability that requires a long time to excite its activity. This characteristic causes the slow exertion of the activity effect and the low early strength of concrete prepared by it, significantly limiting the development of fly ash concrete (FAC).

Two main divisible methods of FA activation are physical activation and chemical activation. Physical activation uses physical means to reduce the size of FA particles, thereby increasing the fly ash specific surface area and the reaction total surface to increase the activity [14,15,16,17,18,19,20]. Chemical activation is an exciting way to promote the release of active components in FA particles by destroying the vitreous structure of the FA shell with acid, alkaline, salt, and other chemical reagents [21,22,23,24,25,26,27,28,29]. However, the physical grinding method consumes a significant amount of energy, and the FA activity improvement effect is limited. The chemical excitation method is more efficient than the physical grinding method, but acid excitation causes severe damage to containers and is inconvenient to operate. Alkali excitation quickly destroys the strength and durability of concrete. Salt excitation causes ion erosion of concrete, resulting in the corrosion of steel bars [30]. Therefore, to realize the green production of FAC and avoid the adverse effects of the introduced activator factors on the durability of concrete, it is necessary to find a new way to stimulate FA’s activity effectively.

Alkaline electrolytic water (AEW) is a kind of highly active water produced by electrolysis with a certain concentration of electrolytes, which is located at the cathode of an electrolytic device [31]. In the electrolysis process, the H-O ionic bond length increases, the bond angle increases, and the attraction between water molecules decreases, leading to high water activity. Consequently, some H-O ionic bonds open, and the water changes into small molecular groups with six molecular groups, which have intense infiltration and penetration ability [32]. At present, these excellent properties of AEW are mainly being used in medical and health care, food, and chemical processing [33,34,35], whereas the applied material research in the concrete industry is still in the preliminary stage [36,37,38]. Therefore, the innovation of this experiment was to use a K_2_CO_3_ electrolyte solution to prepare AEW as concrete mixing water. In addition to high interface permeability, K_2_CO_3_ also had the advantages of low price, stable electrolytic characteristics at low temperature, and high solubility in water, which provide feasibility for promoting cement hydration, stimulating early activity of FA, and preparing green high-performance FAC.

In this work, FA cement-based materials were prepared with ordinary tap water as the blank control group, and fly ash cement-based materials prepared with electrolyzed water with a pH of 9.5, 10.5, and 11.5 were studied. The action mechanism of AEW on FA cement-based materials was observed with microstructure and composition analysis. Furthermore, the effectiveness of AEW in activating FA activity was verified through macroscopic experiments, and the influence of AEW on various properties of FA cement-based materials was analyzed.

## 2. Experimental Program

### 2.1. Raw Materials

In this study, ordinary Portland cement (OPC P·O42.5) produced by Shandong Mountain Aluminum Cement Corp., Ltd. (Qingdao, China) and Grade-II fly ash (FA) produced by Huadian Corp., Ltd. (Weifang, China) were used. The chemical compositions of OPC and FA were characterized using an X-ray fluorescence spectrometer (XRF, XRF-1800 type, Shimadzu Corp., Kyoto, Japan), as shown in Table 1. Natural river sand from Qingdao with a fineness modulus of 2.53 was selected as the fine aggregate, and natural granite gravel with a continuously graded grain size distribution of 5–31.5 mm was selected as the coarse aggregate. The main mineral composition (%) of coarse aggregates produced in Qingdao, China, is potassium feldspar (44%), plagioclase (24%), and quartz (29%). The performance index of the aggregate is shown in Table 2 and Table 3. In addition, the Shandong Institute of Building Science (Jinan, China) created a polycarboxylic acid superplasticizer (NC-J). Ordinary water (OW) available in Qingdao was used, and its pH value was 7.6.

### 2.2. Preparation Procedures of AEW

**Figure 1:**
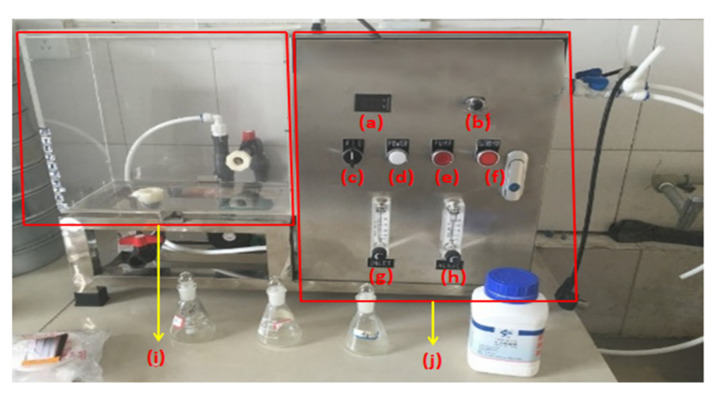
Strong alkalinity full-automatic electrolyzed experimental water machine: (**a**) current and voltage display screen; (**b**) current and voltage adjustment button; (**c**) total power supply; (**d**) power indicator lamp; (**e**) water pump power supply; (**f**) electrolytic power supply; (**g**) total water output adjustment button; (**h**) adjustment button for water output of AEW; (**i**) container; (**j**) electrolyzer.

The electrolysis equipment using a customized, robust alkaline full-automatic electrolyzed water experimental machine (BX-SQJ type, Boxin Corp., Yantai, China), as shown in Figure 1.

**Figure 2:**
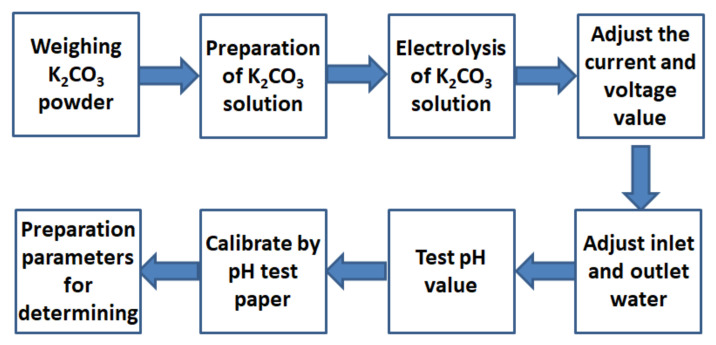
Preparation steps of AEW.

The AEW preparation process adopted the trial production method. The performance of AEW was characterized by the hydrogen ion activity (pH) and oxidation-reduction potential (ORP) value of the solution. The pH value of the electrolytic water increased with electrolyte concentration, voltage, and current and decreased with water inflow and outflow. After the ORP value reached about 200 mV and the conductivity reached about 2700 μs/m, the pH value of AEW was tested with a pH tester and proofread with a pH test paper. After many trials, we satisfactorily established the preparation parameters for AEW with different pH values. Figure 2 gives the preparation steps and the preparation parameters in Table 4.

### 2.3. Design of Experiment

In the functional property test of the paste, the cement dosage was 500 g and the fly ash dosages were 0, 15, 30, and 45%; in turn, they replaced the cement to prepare the OW paste and the AEW paste.

See Table 5 for the mix ratio of the mechanical property test of the fly ash mortar.

The concrete performance test was conducted to prepare the FAC with strength grade C40 based on the best pH value (11.5) of the AEW. See Table 6 for the mix ratio.

### 2.4. Experimental Methods

#### 2.4.1. Hydration Heat Analysis

The hydration heat analysis instrument (TAM Air Type, Lauraie Corp., Shanghai, China) was an eight-channel microcalorimeter. The cement’s hydration heat in the OW and the three kinds of AEW hydration environments were obtained within 72 h by measuring the temperature change of the cement hydration in the calorimeter in a stable temperature environment via the direct method.

#### 2.4.2. XRD Analysis

In the XRD test, under the condition of ensuring a water–cement ratio (W/C) of 0.5, the cement paste with 0%, 15%, 30%, and 45% FA was prepared in turn and then put into the plastic test mold for standard curing for 24 h. Then the mold was removed and it was placed in a curing pool for water cultivation. After three days of aging, the test block was placed in an oven to dry at 60 °C for 5 h and then ground into powder by a powder prototyping machine. Finally, the powder sample was put into an X-ray diffractometer (D9 Advanced Type, Bruker Corp., Karlsruhe, Germany), and the parameters of a voltage of 45 kV, current of 50 mA, 2-theta scanning range of 5–60°, step width of 0.02, and residence time of 0.05 s were set to obtain the diffraction pattern.

#### 2.4.3. Differential Thermal Analysis

The instrument used in the DTA test was an HS-CR-1 type, Hesheng Corp., Shanghai, China. The process of sample preparation was the same as that for XRD analysis. Then the powdered sample was placed in the sample heating seat, and the heating temperature was set to 800 °C and the heating rate was set to 10 °C/min.

#### 2.4.4. SEM Observation

SEM analysis was conducted with a scanning electron microscope (JSM-7500F type, JEOL Corps., Ltd., Tokyo, Japan) to observe the microscopic images of pure cement and 30% FA samples prepared with four kinds of water at 7 days and 28 days of age.

#### 2.4.5. Macroscopic Property Test Methods

**Figure 3:**
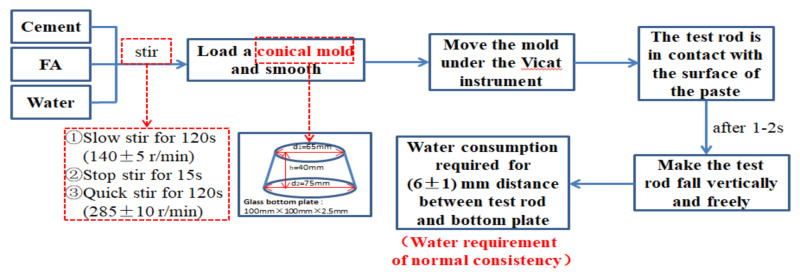
Test process of water requirement for normal consistency.

**Figure 4:**
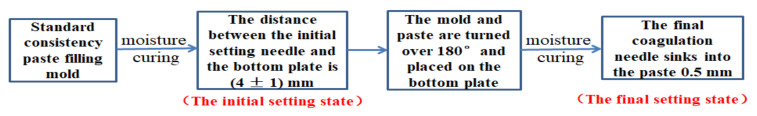
Initial setting state and final setting state.

The working of paste refers to the determination method specified in “Test Method for Water Requirements of Normal Consistency, Setting Time and Stability of Cement Standard Consistency” (GB/T1346-2011, China). The experimental instrument was a standard Vicat instrument (including test rod, initial setting needle, and final setting needle). The test process of water requirements for normal consistency are shown in Figure 3. Initial setting time and final setting time are the time between adding water to the cement paste in the initial setting state and final setting state, respectively, as shown in Figure 4.

The mortar mechanical strength testing was conducted according to the specifications in “Cement Mortar Strength Test Method (ISO Method)” (GB/T17671-1999, China). The mortar was placed in a 40 mm × 40 mm × 160 mm mold in a controlled testing room (T = 20 + 2 °C, RH ≥ 95%) and was cured for 3, 7, 14, and 28 days. The compressive strength of the samples was then determined.

The method of carbonation resistance test and chloride ion penetration resistance of concrete test were conducted according to “Standard for Test Methods of Long-term Performance and Durability of Ordinary Concrete” (GB/T 50082-2009, China), and the size of the carbonization test block was 100 mm × 100 mm × 400 mm. A chloride ion penetration test was evaluated via the rapid chloride ion mobility coefficient method. The calculation formula for the chloride ion mobility coefficient (D_RCM_) is as follows:

(1) $$D_{RCM}=\frac{0.0239(273+T)L}{(U-2)t}(X_{d}-0.0238\sqrt{\frac{(273+T)LX_{d}}{U-2}})$$

In the formula, U is the absolute value (V) of the voltage used, T is the average value of the initial temperature and the end temperature of the anode solution, L is the thickness of the specimen (mm), X_d_ is the average value of chloride ion permeability (mm); and t is the duration of the test (h).

## 3. Results and Discussions

### 3.1. Hydration Heat Analysis

**Figure 5:**
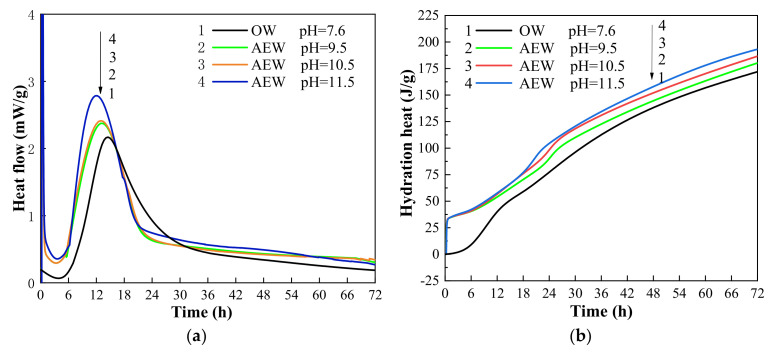
Test chart: (**a**) hydration heat flow diagram; (**b**) hydration heat diagram.

Hydration heat analysis is a method to characterize the hydration process by recording the heat flow in the hydration process of cement. Figure 5 shows the hydration heat flow diagrams (a) and (b) of cement in OW and AEW hydration environments within 72 h. In the figure, OW pH = 7.6 refers to ordinary water with a pH value of 7.6, AEW pH = 9.5 refers to alkaline electrolytic water with a pH value of 9.5, AEW pH = 10.5 refers to alkaline electrolytic water with a pH value of 10.5, and AEW pH = 11.5 refers to alkaline electrolytic water with a pH value of 11.5.

Figure 5a characterizes the real-time heat release of cement hydration. As can be seen from the figure, the induction period of cement hydration in four kinds of water environments was about 6 h, and the corresponding hydration acceleration period was between 6 h and 13 h. However, the time and heat flux corresponding to the peak hydration rate in different environments were different. The peak hydration rate in three kinds of AEW environments with different pH was earlier than that in an ordinary water paste (OWP) environment, and the peak of hydration rate continuously advanced with the increasing pH. Among them, the hydration peaks corresponding to AEW with a pH of 9.5 and 10.5 had a slight difference, and they were 40 min and 64 min earlier than those of OW, respectively. However, the AEW at a pH of 11.5 was 3 h earlier than the typical water environment, and the real-time heat release rate at the peak was 32.2% higher than that in the ordinary water mortar environment. Figure 5b shows the accumulated heat release under a hydration environment. The figure indicates that the hydration heat release of cement in the AEW environment rose linearly within 1 h of hydration. In addition, the hydration heat released in the whole hydration process in the AEW environment was higher than that in OW. Therefore, the experimental results show that AEW promoted the hydration process and hydration degree of cement and released more hydration heat, and the significant effect increased with the increase in the pH value of AEW. The reasons for this phenomenon are as follows: AEW effectively dispersed cement particles, increased the contact surface between water molecules and cement particles, and improved the hydration reaction rate before the hydration reaction. When the hydration reaction was going on, the ionic and high permeability made AEW penetrate the cement particles quickly, which accelerated the hydration process. At the same time, the hydration products wrapped around the surface of cement particles and AEW penetrated the hydration product wrapping layer to improve the effectiveness of the hydration reaction. In addition, the higher the pH value, the higher the concentration of OH^−^ ions in the AEW environment and the lower the activation energy of the cement reaction, thus increasing the hydration rate of cement in the pore solution and speeding up the hydration process [39].

### 3.2. Hydration Product Analysis

#### 3.2.1. XRD Analysis

**Figure 6:**
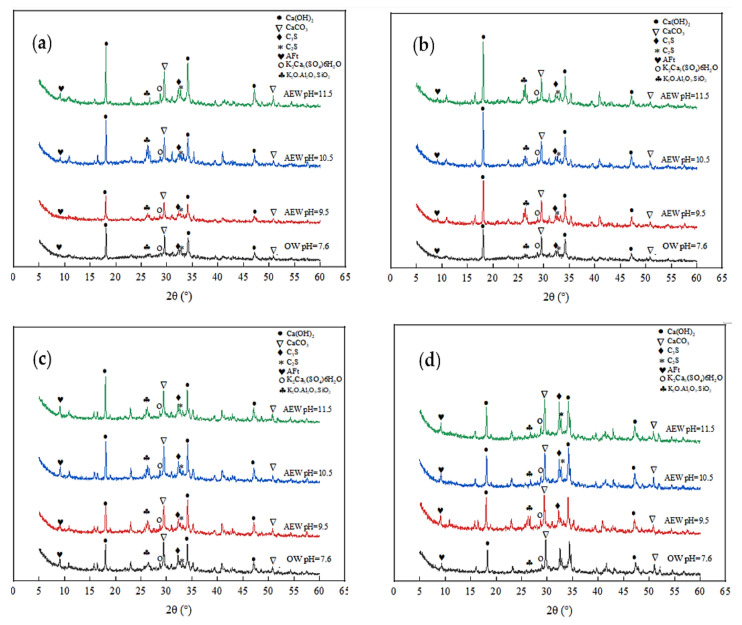
Diffraction pattern of XRD at 7 d: (**a**) FA = 0; (**b**) FA = 15%; (**c**) FA = 30%; (**d**) FA = 45%.

**Figure 7:**
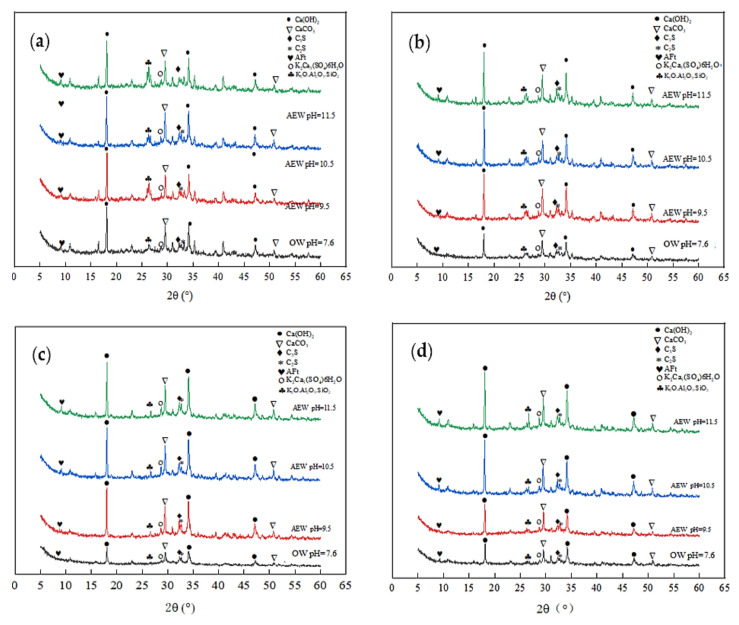
Diffraction pattern of XRD at 28 d: (**a**) FA = 0; (**b**) FA = 15%; (**c**) FA = 30%; (**d**) FA = 45%.

The XRD test quantitatively analyzed the hydration products of different series of pastes at different ages and reasonably explained the development process of macroscopic strength. For example, Figure 6 and Figure 7 show the diffraction patterns of pulp with other FA replacements at 7 and 28 days of age, respectively.

Figure 6 shows that at the age of 7 days, for the same amount of FA, the diffraction intensity of ettringite (AFt) and Ca(OH)_2_ in the AEW test group was more significant than that in the OW test group, and the diffraction peak increased with increasing AEW pH, which shows that AEW promoted the hydration reaction of the cement and produced more C-S-H gel, AFt phase, and Ca(OH)_2_ phase. Besides SiO_2_ and CaCO_3_, the AEW fly ash paste (FAP) contained significant amounts of polycalcium potassium gypsum (K_2_Ca_5_(SO_4_)_6_) and potassium feldspar (K_2_O·Al_2_O_3_·SiO_2_), which is different than the OW FAP tests. With the FA substitution ratio increase, the diffraction peaks of polycalcium potassium gypsum and potassium feldspar, the ettringite peaks, and the hydrated calcium sulphoaluminate peaks of the alkaline electrolyzed water paste with different pH values gradually increased. This effect shows that potassium hydroxide (KOH) in high alkaline electrolytic water reacted with CaO·Al_2_O_3_·SiO_2_ in cement and active Al_2_O_3_ and SiO_2_ in fly ash to form a certain amount of polycalcium potassium gypsum and potassium feldspar in the cement’s early hydration stage, which yielded a positive effect on improving the strength of mortar or concrete.

As shown in Figure 7, when the FA replacement increased gradually from 0% to 45%, the diffraction peaks of the shared water test group weakened, whereas the peaks from the other three AEW test groups remained unchanged. This observation also shows that the effect of the FA replacement on cement hydration in the OW experimental group was far more extensive than that in the AEW experimental group, which further demonstrates AEW’s effectiveness in activating FA activity from the side.

#### 3.2.2. Differential Thermal Analysis

**Figure 8:**
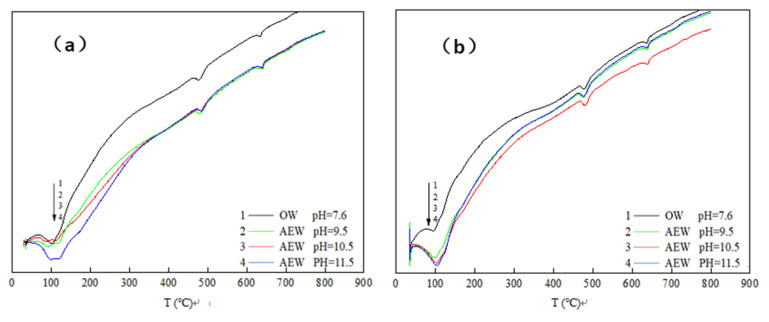

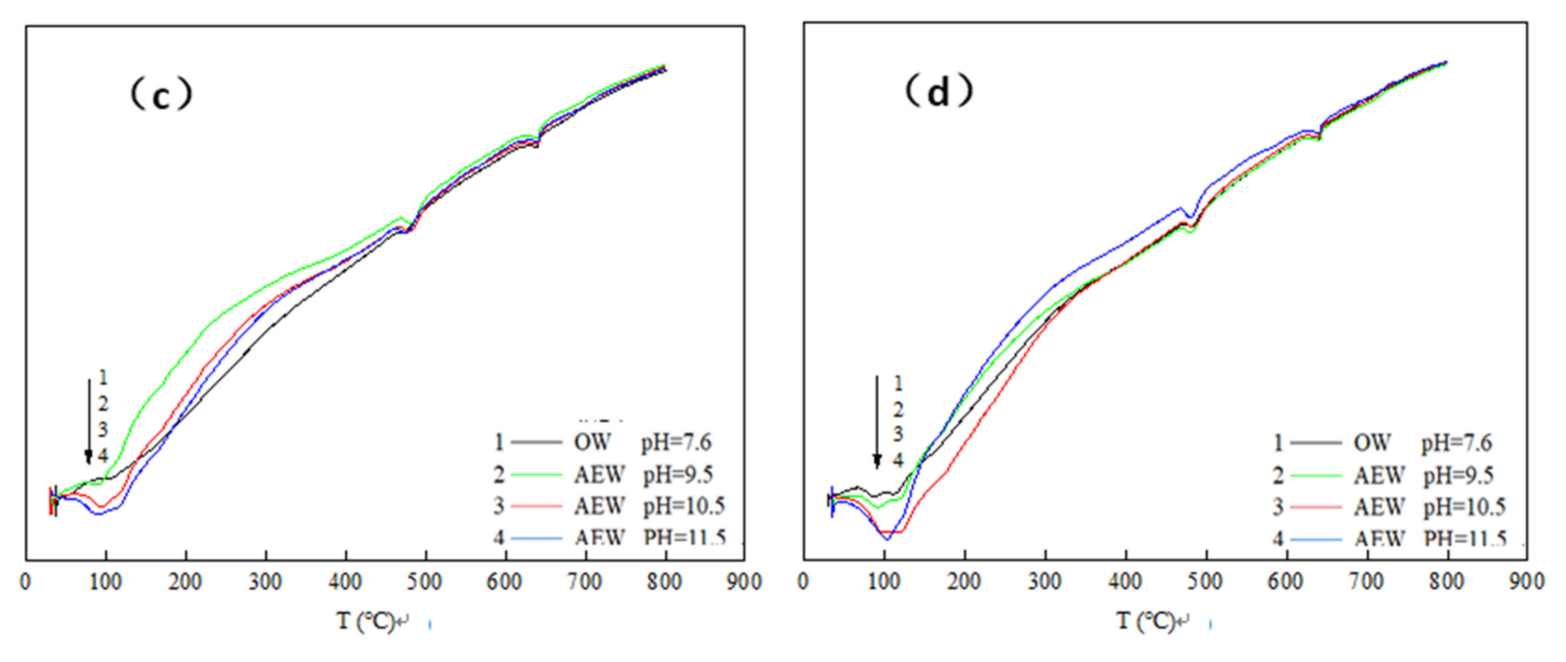
DTA curve of cement paste at 3d: (**a**) FA = 0; (**b**) FA = 15%; (**c**) FA = 30%; (**d**) FA = 45%.

Differential thermal analysis (DTA) characterized the hydration degree and hydration process of cement concrete products. Figure 8 shows the differential thermal analysis map for the paste with different FA replacements prepared with four kinds of water after hydration for three days.

**Figure 9:**
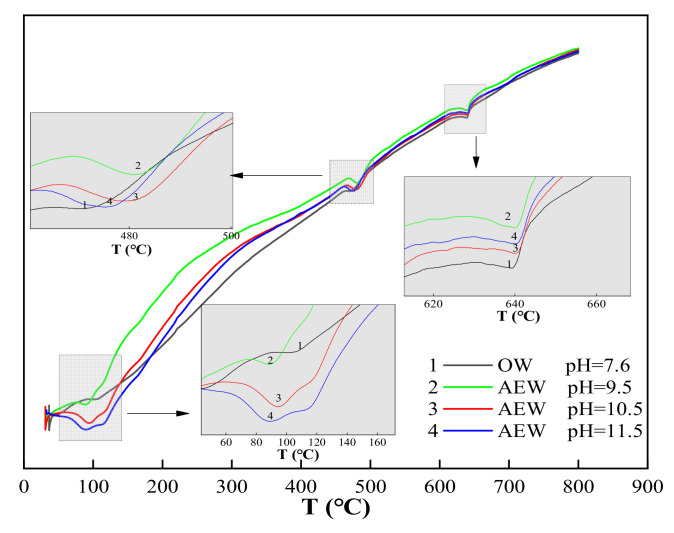
Detailed analysis diagram of Figure 8c.

We took Figure 8c (DTA curve when the FA replacement ratio was 30%) as an example to analyze the DTA curve and obtained a detailed analysis diagram, as shown in Figure 9.

In Figure 9, is can be seen that there were three endothermic peaks in the whole test process of cement paste powder. The first endothermic peak was at around 100 °C, which was the water loss process of C-S-H gel and calcium vanadate. The second endothermic peak was at about 470–490 °C, which was the thermal decomposition process of Ca(OH)_2_. Finally, the third endothermic peak was at around 645 °C, which was the thermal decomposition process of CaCO_3_. Comparing the three endothermic peaks, we observed the following:

At the 100 °C temperature, the endothermic peak of OWP was not apparent; the peak depth of the experimental group with a pH of 9.5 increased prominently; the endothermic peak of the experimental group with a pH of 10.5 deepened further; the endothermic peak of the experimental group with a pH of 11.5 both widened and deepened, indicating that AEW promoted the formation of hydration products with an increased AEW pH value, and the appearance of C-S-H gel and AFt increased. When the temperature rose to 470~490 °C, the endothermic peak for the ordinary water test group was gentle: The endothermic peak with a pH of 9.5 was deepened and widened, and the endothermic peaks of the test groups with a pH of 10.5 and 11.5 were further broadened. Hence, we concluded that AEW promoted the formation of the hydration product Ca(OH)_2_ when heated to 645 °C. The order of areas for the respective CaCO_3_ endothermic peaks in each experimental group was pH = 9.5 AEW > pH = 11.5 AEW > pH = 7.6 AEW > pH = 10.5 AEW. The law of endothermic peaks differed for the first two peaks because AEW promoted the hydration process and inhibited simultaneous carbonization. The superposition of positive and negative reaction effects made the CaCO_3_ content in the experimental group different from the above two hydration products.

From the microstructural point of view, the differential thermal test also showed that AEW can significantly promote the hydration process and hydration reaction degree of cement, which is consistent with the results of the XRD test.

### 3.3. SEM Micromorphology

**Figure 10:**
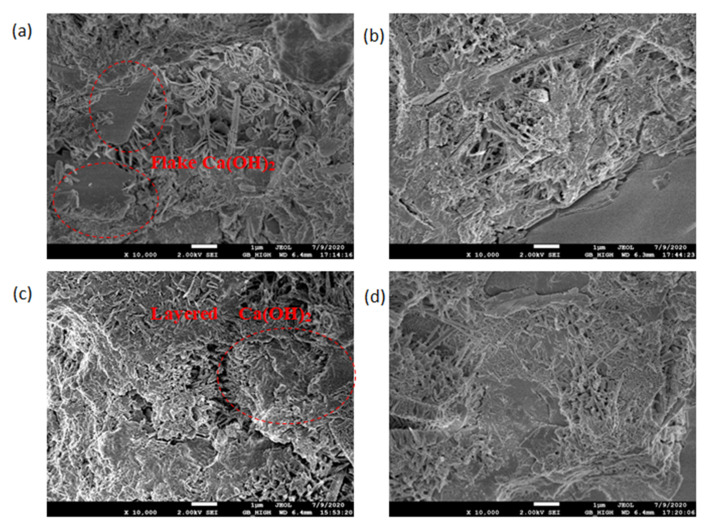
SEM diagram of pure cement paste (FA replacement ratio is 0%) at 7 days of age: (**a**) pH = 7.6 OW; (**b**) pH = 9.5 AEW; (**c**) pH = 10.5 AEW; (**d**) pH = 11.5 AEW.

**Figure 11:**
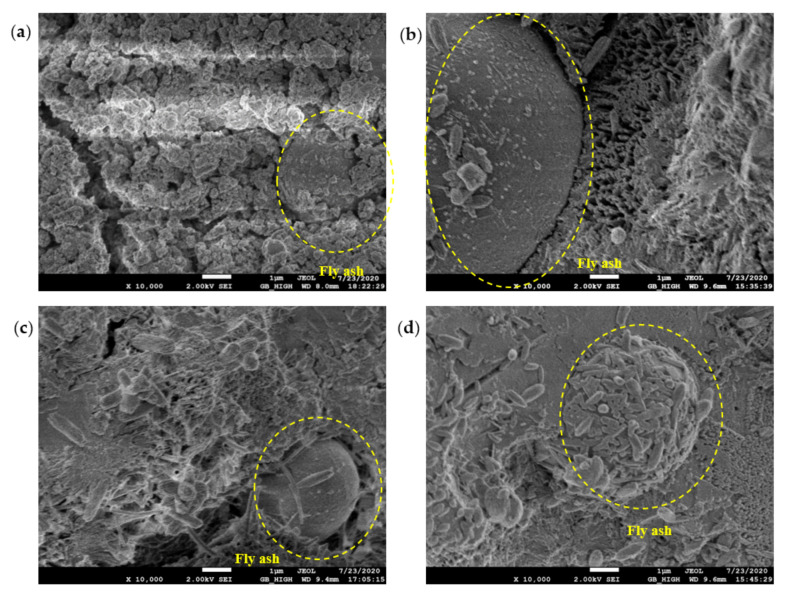
SEM diagram of pure cement paste (FA replacement ratio is 30%) at 7 days of age: (**a**) pH = 7.6 OW; (**b**) pH = 9.5 AEW; (**c**) pH = 10.5 AEW; (**d**) pH = 11.5 AEW.

**Figure 12:**
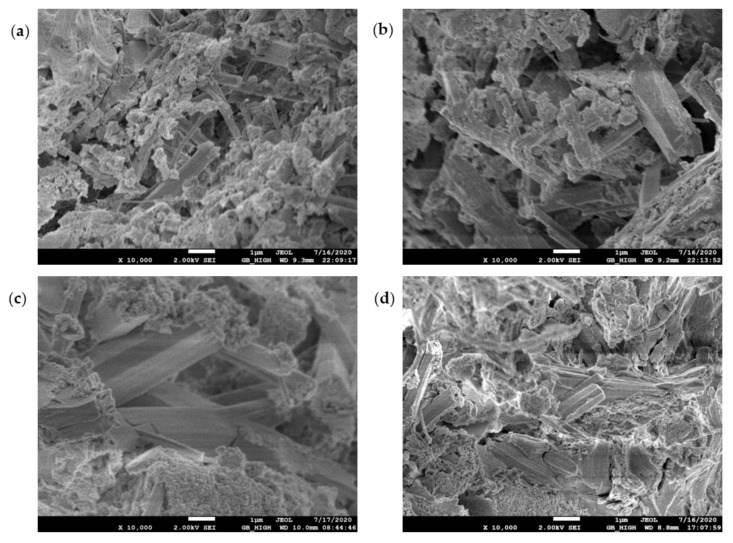
SEM diagram of pure cement paste (FA replacement ratio is 0%) at 28 days of age: (**a**) pH = 7.6 OW; (**b**) pH = 9.5 AEW; (**c**) pH = 10.5 AEW; (**d**) pH = 11.5 AEW.

**Figure 13:**
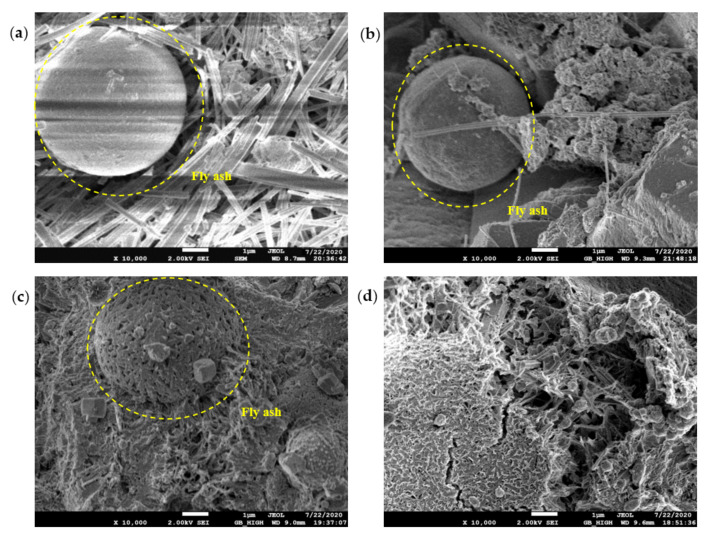
SEM diagram of pure cement paste (FA replacement ratio is 30%) at 28 days of age: (**a**) pH = 7.6 OW; (**b**) pH = 9.5 AEW; (**c**) pH = 10.5 AEW; (**d**) pH = 11.5 AEW.

The SEM test observed the morphology, loading, quantity, pores, and cracks of the cement hydrate and verified the macroscopic strength of the cement mixture. Figure 10, Figure 11, Figure 12 and Figure 13 are SEM micrographs of the FA cement paste with 0% and 30% FA replacement at 7 days and 28 days of age, respectively.

As shown in Figure 10, with increasing pH, the distribution of the hydration products in the cement paste changed from dispersion to gradual connection, and its structure gradually became denser. In Figure 10a, the Ca(OH)_2_ flake produced with the OW pure water sample at the age of 7 days was thin and irregular in shape, and had not yet gained a hexagonal plate shape. The needle-shaped ettringite produced was fine, and the inter-connection between the hydration products was poor. In Figure 10b, the ettringite in the hydration products of the AEW cement slurry sample with a pH of 9.5 overlapped each other to form a network structure. The Ca(OH)_2_ in the hydration product of the AEW sample with a pH of 10.5, as seen in Figure 10c, was lamellar, and closely connected with ettringite. As seen in Figure 10d, the hydration product at a pH of 11.5 was more compact, the macropores were reduced, and the overlapping of the lamellar Ca(OH)_2_ was closer.

Figure 11 shows morphological observations of the hydration products around the FA particles. It was observed that there were markedly more hydration products in the AEW test group than in the OW test group, and with increasing pH, hydration products such as ettringite gradually increased. It is particularly noteworthy that in the micrographs of the samples with a pH value of 11.5, hydration products surrounding the FA particles were formed on their surfaces, suggesting that AEW with a pH value of 11.5 promoted the decomposition of the vitreous shell of the FA particles, stimulated the activity of the FA, and released active components such as SiO_2_ for the hydration reaction.

Figure 12a–d shows that after curing to 28 days of age, with increasing pH, the volume of ettringite increased and gradually presented a dense state, which shows that AEW promoted the growth and thickening of ettringite, which plays a significant role in improving the overall macroscopic strength of cementitious materials.

In the OW sample micrographs shown in Figure 13a, although there was a large amount of ettringite around the FA particles, its glassy shell remained intact. The FA particles shown in Figure 13b were surrounded by ettringite, and ettringite existed on the surface of the shell, suggesting that alkaline electrolysis dissolved its glass shell to release active components and produce hydration products on the surface of the particles. Furthermore, the network structure of the FA particles in Figure 13c was gradually dissolved, and the existence of potassium gypsum was found on the surface of the FA particles, showing that K^+^ in potassium-based AEW may participate in the hydration reaction and form potassium gypsum (K_2_Ca_5_(SO_4_)_6_). Figure 13d shows that the shell of the FA in the AEW sample with a pH of 11.5 was completely dissolved, and a large number of ettringite and gel conjugates were formed after the FA broke, confirming the effectiveness of AEW with a pH of 11.5 in activating FA.

### 3.4. Workability of the Paste

**Figure 14:**
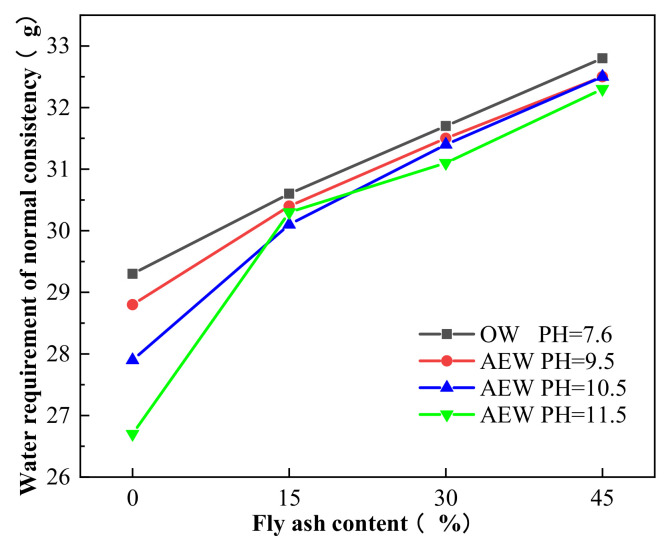
Comparison of the water requirement for a normal consistency of cement paste.

Figure 14 shows the water requirement of the normal consistency of paste with 0%, 15%, 30%, and 45% FA mixed with OW and AEW at different pH.

Figure 14 shows that with an increased FA substitution rate, the water requirement for a normal consistency of cement paste mixed with OW and AEW showed an upward trend. However, for a certain FA substitution ratio, the water requirement for a normal consistency of alkaline electrolyzed water paste (AEWP) with three different pH values was lower than that of OWP, and increased pH value in AEW the water requirement for normal consistency of AEW decreased. For example, when the pH of AEW was 11.5, the water requirement for the normal consistency of pure cement paste was at its lowest, 8.9% lower than that of OW pure cement paste. This result shows that AEW improved the fluidity of cement paste, and when the pH value was 11.5, the fluidity of the cement paste was at its best. This fact, too, was due to the small clustered structure of the water molecules in AEW, which made it easier for the water molecules to adhere to the surface of the cement particles uniformly. Simultaneously, the charged free radicals had the same charge dispersion effect on the agglomerated cement particles, which slowed down the flocculation phenomenon of the cement particles, increased the contact surface between the water molecules and cement FA particles, and made the water molecules play a better lubrication role, thus achieving the effect of the improved fluidity of the cement mixture [39].

**Figure 15:**
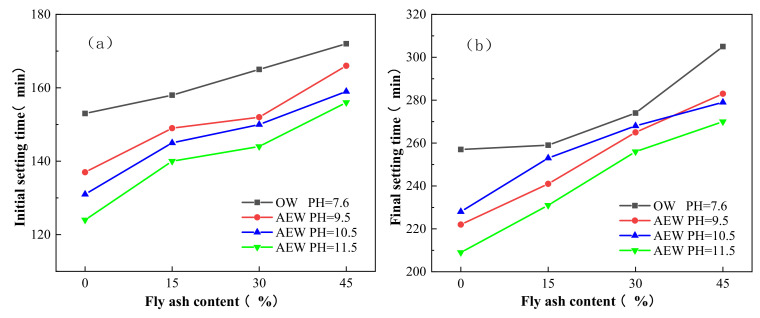
Initial and final setting times of OW and AEW with different pH for different FA replacements: (**a**) Initial setting time; (**b**) Final setting time.

Figure 15 gives the initial and final setting times of OW and AEW with different pH for different FA replacements. In the figure, OW pH = 7.6 refers to the paste prepared with ordinary water at a pH of 7.6. AEW pH = 9.5 refers to the paste prepared with alkaline electrolytic water at a pH of 9.5. AEW pH = 10.5 refers to the paste prepared with alkaline electrolytic water at a pH of 10.5. AEW pH = 11.5 refers to the paste prepared with alkaline electrolytic water at a pH of 11.5.

Figure 15a,b shows that OW’s initial and final setting times and different AEWP increased continuously with the increasing FA substitution rate. Concurrently, the initial and final setting times of the AEW standard consistency paste with varying values of pH were shorter than those of the OW standard consistency paste, and when the pH value of AEW was 11.5, the initial and final setting times of cement paste with the same FA substitution rate were the shortest. Specifically, the initial setting time of the cement paste with a pH of 11. 5AEW was shortened by 29, 18, 21 and 16 min, and the final setting time was shortened by 48, 28, 18 and 35 min, respectively. In addition, the FA replacement was 0, 15, 30, and 45, compared to the OW control group. Thus, the above data show that AEW can accelerate cement paste’s setting speed and shorten its setting time, which is also favorable evidence that AEW can effectively promote cement hydration.

### 3.5. Mechanical Properties of Mortar

**Figure 16:**
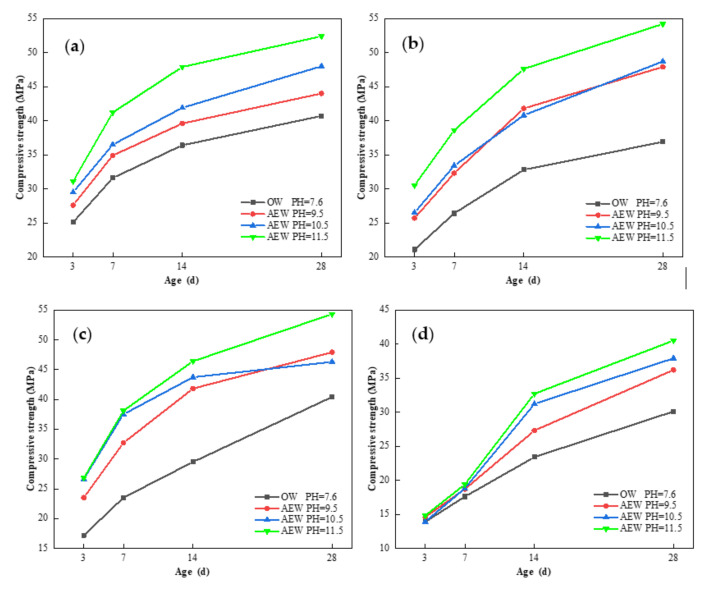
Effect of OW and AEW with different pH values on the compressive strength of FAM: (**a**) FA = 0; (**b**) FA = 15%; (**c**) FA = 30%; (**d**) FA = 45%.

Figure 16 shows the effect of OW and AEW with different pH values on the compressive strength of fly ash mortar (FAM) at 3, 7, 14 and 28 days of age.

It can be seen from Figure 16a–c that when the replacement of FA was low, the compressive strength of alkaline electrolyzed water mortar (AEWM) with different pH values was higher than that of ordinary water mortar (OWM) for all aged samples, among which the compressive strength of AEWM with a pH of 9.5 and 10.5 was very close and in the middle, respectively, and the compressive strength of AEWM with pH 11.5 was the highest. The compressive strength of AEWM with a pH of 11.5 increased by 23.9, 44.5, and 55.8% in three days and by 28.7, 36.9, and 34.4% in 28 days, respectively, compared to that of OWM with identical FA replacements of 0, 15, and 30% of FA. This shows that AEW contributed significantly to the development of the compressive strength of FAM in the early stages and improved the overall strength of FAM in the later stages. Figure 16d shows that when the FA replacement reached 45%, the strength of both OWM and AEWM decreased noticeably at all ages, and the compressive strength value of AEWM after three days’ curing was slightly different than that of OWM. The maximum strength growth rate was only 6.5%, indicating that the higher fly ash content adversely affected the overall strength of mortar, and that AEW played a small role in the initial hydration stage.

In addition, at the age of 28 days, the compressive strength of AEWM with 30% FA and a pH value of 11.5 was 33.4% higher than that of pure cement mortar without FA. This demonstrates that AEW ensured the mechanical properties of the mortar on the premise of increasing the amount of FA that achieved cement reduction and the goal of the efficient utilization of FA.

### 3.6. Durability of Concrete

The durability test for alkaline electrolyzed water concrete (AEWC) was to select the best AEW with a pH value of 11.5 based on the performance test of paste and mortar, to prepare concrete with FA replacements of 0, 15, 30 and 45%, and to take ordinary water concrete (OWC) as the control group to study the carbonization resistance and chloride ion penetration resistance. 

**Figure 17:**
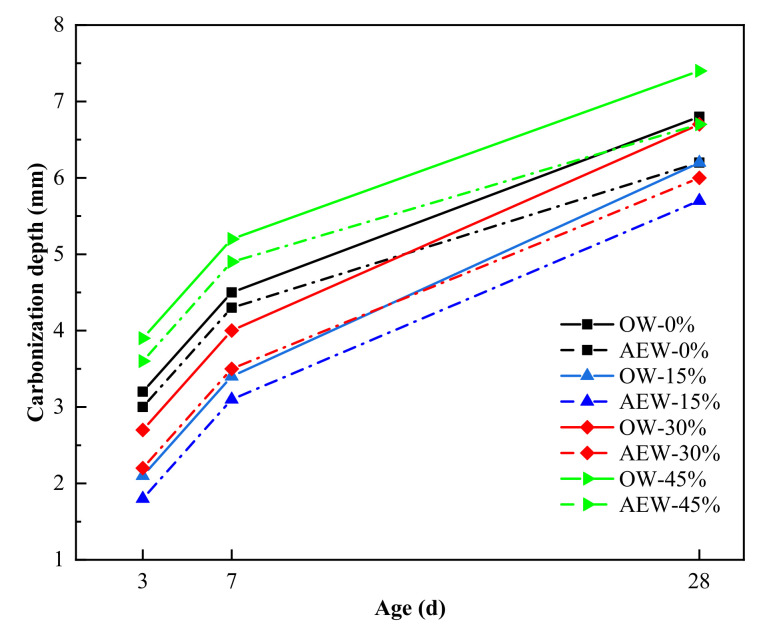
Effect of OW and AEW with a pH value of 11.5 on the carbonation resistance of concrete with different FA replacements at different ages.

Figure 17 shows the carbonation resistance of concrete. In this figure, OW-0% refers to the concrete with 0% fly ash content prepared by ordinary water. AEW-0% refers to the concrete with 0% fly ash content prepared by alkaline electrolytic water. OW-45% refers to the concrete with 45% fly ash content prepared by ordinary water. AEW-45% refers to the concrete with 45% fly ash content prepared by alkaline electrolytic water.

Looking at Figure 17, we see that the carbonation depth and carbonation rate of AEWC were lower than those of OWC. For example, when the FA replacement was 0, 15, 30, and 45%, the carbonation depth of AEWC decreased by 8.7, 8.1, 10.4 and 9.5%, respectively, compared to that of the OWC group with the same FA content. In addition, at 28 days of age, the carbonation depth of AEWC with 45% FA was still less than that of OWC without FA. Thus, AEW reduced the carbonation depth, slowed down the concrete carbonation rate, and maintained good carbonation resistance with high FA replacement. This is because AEW improved the hydration degree of the cementitious materials, and made the hydration products fill the concrete pores more evenly, thus effectively reducing the invasion degree of CO_2_. Concurrently, the alkaline environment of AEW improved the alkali reserve in the concrete structure and kept the alkaline environment in coagulation stable during the carbonation process [40,41], which proved beneficial to improving the concrete carbonation resistance.

**Figure 18:**
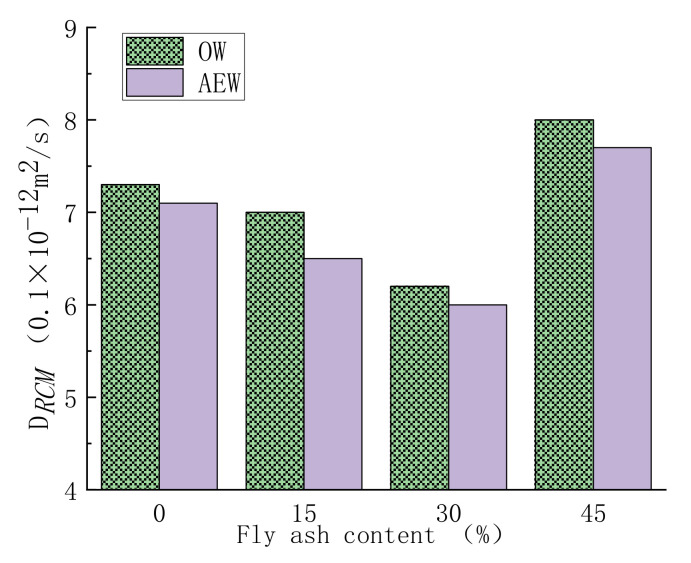
Chloride ion transport coefficient of concrete with OW and AEW with a pH value of 11.5 under different FA replacements.

Figure 18 shows the chloride ion penetration resistance of concrete.

Figure 18 shows concrete’s chloride ion penetration resistance with different FA replacements in the two water environments. The experimental results show that with increasing FA replacement, the D*_RCM_* value of concrete first decreased and then increased markedly, and when the optimum FA replacement was 30%, the D*_RCM_* value was the smallest. At the same time, the D*_RCM_* value of AEWC was less than that of OWC in all mix ratios. The main factor affecting chloride ion transport and diffusion was the structural compactness of concrete [42]. After 28 days of curing, the pozzolanic reaction products of FA filled the internal pores of the concrete, making the structure more compact. However, when the FA replacement was too large, the active components involved in hydration in the cementitious material system decreased, and the FA particles mainly showed a filling effect, which weakened the structure density [43]. In addition, compared with OWC, the positive excitation effect of AEW on FA activity made AEWC less sensitive to FA replacement but still maintained good resistance to chloride ion erosion.

## 4. Conclusions

The influence of alkaline electrolysis water on the cement hydration process was analyzed through hydration heat and differential thermal testing. Using XRD and SEM, we observed the crystal composition and structural morphology of the cement hydration product composition and structure morphology of cement. Microstructural research and the macroscopic experiments on the workability of paste, the mechanical properties of mortar, and various properties of concrete were studied, with the results obtained leading us to make the following conclusions.

1. AEW promoted the hydration process and hydration degree of cement and released more hydration heat, and their influence effectively increased with the increased pH value of AEW.
2. AEW increased the formation of C-S-H gel, ettringite, and the Ca(OH)_2_ phase in cement hydration products. In addition, unlike OW, potassium hydroxide (KOH) in potassium-based AEW reacted with active minerals Al_2_O_3_ and SiO_2_ in cement and FA to form certain amounts of polycalcium potassium gypsum and potassium feldspar, which proved beneficial to the improvement of macroscopic strength.
3. SEM observations showed that the higher the pH value of AEW samples, the denser the structure of the hydration products produced, and AEW with a pH value of 11.5 had a pronounced decomposition effect on the vitreous shell of FA, which better released SiO_2_ and other active components for hydration reaction, proving the feasibility of AEW to activate FA activity.
4. The charge and small molecules of AEW effectively improved the functional properties of the paste, reduced the water requirement for a normal consistency, and shortened the initial setting time and final setting time, which was direct evidence that AEW promotes cement hydration. AEW also effectively improved the early and late strength of FA mortar and maintained excellent mechanical properties when the FA content was increased to 30%, thus achieving cement reduction. In addition, the hydration promotion effect of AEW and its alkali reserve reduced the carbonation rate and carbonation depth of concrete and improved concrete’s chloride ion penetration resistance.

## Figures and Tables

**Table 1:** Chemical composition of OPC and FA by XRF, wt.%.

**Constituent**	CaO	SiO_2_	Al_2_O_3_	Fe_2_O_3_	SO_3_	MgO	Na_2_O	K_2_O	TiO_2_
**OPC**	62.73	17.80	6.38	5.83	2.98	1.94	0.86	0.58	0.52
**FA**	2.41	57.36	28.68	4.29	0.35	1.28	0.73	0.83	1.92

**Table 2:** Performance index of natural fine aggregate.

Fineness Module	Specifications	Stacking Density (kg/m^3^)	Apparent Density (kg/m^3^)	Void Ratio/%	Micro Powder Content/%	Mud Content/%	Crushing Index/%
2.43	Medium sand Ⅱ class	1450	2590	40	1.0	0.7	13

**Table 3:** Performance index of natural coarse aggregate.

Water Absorption/%	Moisture Content/%	Content of Needle-like Particles/%	Crushing Index/%	Stacking Density (kg/m^3^)	Apparent Density (kg/m^3^)
1.7	0.42	4.05	11.2	1460	2510

**Table 4:** Preparation parameters of AEW with different pH values.

PH Value	ORP Value	K_2_CO_3_ Solution Concentration (%)	Total Inflow (L/h)	Alkaline Water Inflow (L/h)	Current (mA)	Voltage (V)
9.5	224	0.05	40	20	0.6	10.1
10.5	207	0.08	40	20	1.0	18.7
11.5	200	0.10	40	20	3.0	25.6

**Table 5:** Mix proportion of the fly ash mortar (kg/m^3^).

Types of Water	PH Value	Water	Cement	Fly Ash	Sand	Water Reducer
OW	7.6	225	450	0	1350	5.4
OW	7.6		382.5	67.5		
OW	7.6		315	135		
OW	7.6		247.5	202.5		
AEW	9.5		450	0		
AEW	9.5		382.5	67.5		
AEW	9.5		315	135		
AEW	9.5		247.5	202.5		
AEW	10.5		450	0		
AEW	10.5		382.5	67.5		
AEW	10.5		315	135		
AEW	10.5		247.5	202.5		
AEW	11.5		450	0		
AEW	11.5		382.5	67.5		
AEW	11.5		315	135		
AEW	11.5		247.5	202.5		

**Table 6:** Mix proportion of the FAC (kg/m^3^).

Types of Water	Water	Cement	Fly Ash	River Sand	Gravel	Water Reducer	W/C
OW	163	440	0	843	861	8.8	0.37
AEW		440	0				
OW		374	66				
AEW		374	66				
OW		308	132				
AEW		308	132				
OW		242	198				
AEW		242	198				

## Data Availability

Since the experiment was completed with the support of Qingdao Agricultural University and Qingdao University of Technology, the data used to support the results of this study are available from the responsible person and the author upon request.

## Abbreviations

The following abbreviations were adopted in this manuscript:

AEW	alkaline electrolyzed water
AEWC	alkaline electrolyzed water concrete
AEWM	alkaline electrolyzed water mortar
AEWP	alkaline electrolyzed water paste
FA	fly ash
FAC	fly ash concrete
FAM	fly ash mortar
FAP	fly ash paste
OW	ordinary water
OWC	ordinary water concrete
OWM	ordinary water mortar
OWP	ordinary water paste
